# Automatic Change Detection of Human Attractiveness: Comparing Visual and Auditory Perception

**DOI:** 10.3390/brainsci15111226

**Published:** 2025-11-15

**Authors:** Meng Liu, Jin Gao, Werner Sommer, Weijun Li

**Affiliations:** 1 Department of Psychology, Liaoning Normal University, Dalian 116029, China; 2 Institute of Psychological and Brain Sciences, Liaoning Normal University, Dalian 116029, China; 3 Institut für Psychologie, Humboldt-Universität zu Berlin, 10117 Berlin, Germany; werner.sommer@cms.hu-berlin.de

**Keywords:** attractiveness, change detection, mismatch, ERPs, negativity bias

## Abstract

Background/Objectives: Change detection of social cues across individuals plays an important role in human interaction. Methods: Here we investigated the automatic change detection of facial and vocal attractiveness in 19 female participants by recording event-related potentials (ERPs). We adopted a ‘deviant-standard-reverse’ oddball paradigm where high- or low-attractive items were embedded as deviants in a sequence of opposite attractive standard stimuli. Results: Both high- and low-attractive faces and voices elicited mismatch negativities (MMNs). Furthermore, low-attractive versus high-attractive items induced larger mismatch negativities in the voice condition but larger P3 amplitudes in the face condition. Conclusions: These data indicate that attractiveness can be automatically detected but that differences exist between facial and vocal attractiveness processing. Generally, change detection seems to work better for unattractive than attractive information, possibly in line with a negativity bias.

## 1. Introduction

Detecting and coping appropriately with environmental novelty and change is highly important. In humans, responding to environmental change has been investigated in oddball paradigms, where rare stimuli unpredictably occur on a background of frequent standard stimuli. These studies show that change (oddball) detection takes place not only with respect to physical stimulus features (e.g., color, line orientation, motion direction, spatial frequency, intensity, or pitch) but also along more complex and socially relevant dimensions, such as posture [1]. Interestingly, some studies demonstrated that unpleasant valence images can produce larger P1 amplitudes than pleasant and neutral images [2]. These findings are consistent with the considerable literature about negativity bias (or negative superiority effect), suggesting that, as compared to positive stimuli, negative stimuli are processed more efficiently [3]. Physical attractiveness and unattractiveness are important dimensions of social relevance, involving aspects of facial and vocal attractiveness, and play a central role in human interaction [4,5]. In the present study, we used ERP recordings to assess the automaticity of detecting facial and vocal attractiveness and whether it entails a negativity bias.

There is extensive evidence in support of the evolutionary functions of attractiveness and its role in daily life. It is generally agreed that attractiveness plays a central role in the assessment of mate value [4,6]. The perception of attractiveness may help to select high-quality mates and transmit one’s genes to the next generation [7]. However, attractiveness also plays an important role in other social contexts. Thus, attractive individuals tend to be more successful in friendship formation, job interviews, political elections, and they earn more money [8,9]. In addition, attractive people are usually associated with positive personality traits, observed for both visual appearance and vocal properties [10,11,12,13].

Research on attractiveness perception has focused on facial attractiveness, which can be perceived even when faces are masked or presented rapidly [14,15] and may direct participants’ attention in the absence of consciousness [16]. EEG studies show that attractiveness may affect the early perceptual and cognitive processing of faces, as indicated by the N170 component, which reflects structural face representations, and by the anterior P2 component (120–220 ms), suggesting a fast attentional bias to attractive opposite-sex faces [5,17]. Facial attractiveness also affects later cognitive processes, as reflected in the centro-parietal late positive complex (LPC), linked to motivated attention [5,18].

Although the overall attractiveness of a person substantially relates to visual cues from face and body [19], it is also influenced by her or his voice [20]. Indeed, both facial and vocal attractiveness have been linked to traits indicating sex hormone levels and health [21], and attractiveness judgments often co-vary across modalities [22]. Previous studies also suggested that averageness and sexual dimorphism are common factors influencing facial and vocal attractiveness judgments [23]. However, compared with the relatively extensive research on facial attractiveness, most studies on vocal attractiveness have been conducted in the evolutionary domain, suggesting that non-verbal voice features, such as pitch, provide ecologically relevant information about the speaker [20]. Only a few studies have explored the neurophysiological mechanism of perceiving vocal attractiveness [5,24,25,26]. For example, Zhang et al. [26] administered an implicit tone detection task and an explicit attractiveness judgment task with short voice samples, using the ERP technique. In both tasks N1 amplitudes were larger for attractive than unattractive voices, whereas attractive voices elicited larger LPC amplitudes only in the explicit task. Hence, vocal attractiveness processing during early stages appears to be rapid and mandatory, whereas during later elaborated stages, vocal attractiveness is processed strategically and is aesthetics-based, requiring attentional resources.

Of note, the research on facial and vocal attractiveness mentioned above mainly adopted explicit tasks. Although the perception of attractiveness should occur automatically at early stages of attention, before any significant cognitive investment is made [27], there is little research involving pre-attentive conditions. In the ERP, fast pre-attentive change detection can be indicated by the mismatch negativity (MMN) and P3 components [28]. The auditory MMN (aMMN) with a frontocentral scalp distribution is usually observed around 100–250 ms after the onset of a deviant sound [29], and the visual MMN (vMMN) appears 100–300 ms after visual deviants and shows a posterior scalp distribution [30]. The parietally distributed P3 component, elicited when events require the updating of stimulus representations held in working memory by involuntary attention capture or conscious deviance detection, usually peaks around 300 ms [31]. The P3 may occur in implicit tasks but is usually much more pronounced in explicit tasks.

Although previous studies have found that people can automatically recognize some high-level face features (e.g., expression and gender) [32,33], few studies have focused on the automatic processing characteristics of human attractiveness information. Using EEG, some of these studies found that face-sensitive visual areas can discriminate implicitly and rapidly between levels of facial attractiveness [34,35] and that attractive faces can be processed automatically by both males and females [27]. However, other research indicates that facial attractiveness processing is not mandatory, since the attractiveness-related modulations of brain responses were only a trend during a gender decision task [36]. Hence, there may be early automatic processes of attractiveness detection and later controlled processes. It is even less clear whether vocal attractiveness information can be perceived automatically and how this perception compares to facial attractiveness.

A further question of interest is the contrast between high and low attractiveness and how it depends on the facial versus vocal domain. Interestingly, some studies demonstrated that the change detection of emotional valence is more sensitive for negative than positive valence [37,38]. These findings are consistent with the considerable literature about the negativity bias (or negative superiority effect), suggesting that, as compared to positive stimuli, negative stimuli are processed more efficiently [3]. This might also hold for unattractiveness as compared to attractiveness.

The present study adopted a deviant-standard reverse oddball paradigm [39], in which probability and attractiveness were manipulated in order to investigate the correlates of implicit facial and vocal attractiveness processing. Our prominent aim was to investigate whether change detection during implicit facial and vocal attractiveness perception is modulated by high versus low attractiveness categories. Specifically, given that automatic change detection processes are facilitated by the emotional quality of faces [40] or vocalizations [41], we also hypothesized that attractiveness differences would induce change detection processes, as indexed by the MMN and P3 components. If change detection is facilitated by unattractive faces or voices (i.e., unattractive stimuli associated with avoidance), we expected increased MMN and P3 amplitudes for unattractive deviants relative to attractive standard stimuli. Also, if change detection is facilitated by attractive cues (i.e., attractive stimuli associated with pleasure and approach), one should expect increased MMN and P3 amplitudes for attractive deviants compared with unattractive standard stimuli. In line with the negativity bias, we expect the effect for unattractive deviants to be more pronounced than for attractive deviants.

## 2. Materials and Methods

### 2.1. Participants

The current study involved a 2 (attractiveness: attractive, unattractive) × 2 (deviance: standard, deviant) × 2 (hemisphere: left, right) within-participants design. A pilot study with 15 participants had shown that the effect size for this interaction was *η_p_*^2^ = 0.46. A priori power analysis conducted via G*Power 3.1.9 [42] showed that 18 participants were required to observe a significant (*α* = 0.05) interaction at 0.80 power. Based on these considerations, twenty-one female undergraduate students participated in the current experiment, receiving 50 RMB as compensation. All of them reported normal or corrected-to-normal vision, no history of hearing or neurological impairments, and were right handed. This study was approved by the Research Ethics Committee of Liaoning Normal University (No. LL2025229). Each participant had given informed consent in accordance with the Declaration of Helsinki before participation. Owing to excessive EEG artifacts, data from two participants were excluded; the final sample included 19 female participants, aged 18 to 24 years (*M* = 21.62 ± 1.3 years). In order to reduce complexity, we only tested female participants with stimuli from male actors.

### 2.2. Materials

The initial pool of facial stimuli consisted of pictures of 30 attractive and 30 unattractive male faces that had been used in a previous study [43]. All faces showed neutral expressions, were taken from a frontal view and with eye gaze directed at the observer. To exclude non-facial information, external features (i.e., hair, ears) were removed and images were cropped to an approximate size of 260 × 300 pixels. Forty female undergraduate students (age range 18–25 years, *M* = 22.38 ± 1.6 years) who did not participate in the ERP experiment validated the 60 faces on a 7-point rating scale (ranging from 1 = very unattractive to 7 = very attractive). The five most attractive (*M* = 5.28) and five most unattractive (*M* = 2.01) faces were selected as stimuli for the ERP experiment. Statistical analysis confirmed significantly higher ratings for attractive than for unattractive faces, *t* (8) = 21.80, *p* < 0.001, and *d* = 13.79. In addition, each of these ten faces was also set with a red dot on the nose as target stimuli for the experimental task.

The initial pool of male vocal stimuli consisted of the 40 attractive and 40 unattractive vowel syllables from a previous study [26]. These syllables, recorded with Adobe Audition software and digitized at 16-bit/44.1 kHz, were provided by professional male speakers in an emotionally neutral prosody. Praat software (http://www.fon.hum.uva.nl/praat/, accessed on 1 October 2017) was used to equalize the duration (400 ms) and normalize the intensity (70 dB) of the syllables. Thirty further female undergraduate students (age range 18–25 years, *M* = 21.26 ± 1.2 years) rated the vocal materials on a 7-point scale (ranging from 1 = very unattractive to 7 = very attractive). From the forty rated syllables, the five most attractive (*M* = 5.09) and five most unattractive (*M* = 2.74) ones were selected as stimuli for the ERP experiment. Statistical analysis confirmed significantly higher ratings for attractive than for unattractive voices, *t* (8) = 17.02, *p* < 0.001, and *d* = 10.76. Furthermore, each of the ten syllables was also mixed with white noise, serving as target stimuli.

### 2.3. Procedure

**Figure 1:**
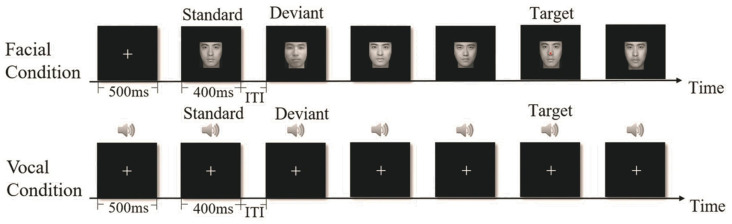
Schematic illustration of the unattractive in attractive Oddball condition for face condition and voice condition. Stimulus duration was 400 ms for both faces and voices; the inter-trial intervals (ITI) varied between 1300 and 1500 ms.

Participants were comfortably seated in an electrically shielded room at a distance of 90 cm from a computer screen. Stimuli were presented on a 24-inch LCD monitor (1920 × 1080 resolution, 60 Hz refresh rate). Each face subtended 4.6 × 5.3 visual degrees. The deviant-standard reverse oddball paradigm was conducted in separate face and voice conditions (see Figure 1), with the order of conditions counterbalanced across participants. Visual stimuli were presented at the center of the screen, and auditory stimuli were presented via headphones at a comfortable listening level. In both conditions, simultaneous appearance of a fixation cross and warning tone (500 ms) at the beginning of each block was followed by a sequence of face or voice stimuli. The duration of each face/voice stimulus was 400 ms, and the inter-trial intervals (ITI) varied between 1.3 and 1.5 s (see Figure 1). Participants were instructed to complete an attractiveness-unrelated task by pressing the space key as quickly as possible whenever they saw a face with red nose (targets) in the face condition or heard a voice mixed with white noise (targets) in the voice condition. Attractive and unattractive stimuli were presented as both standards and deviants to eliminate the differences in the physical properties between faces/voices. Overall, the ERP experiment was composed of four conditions: (1) attractive face standards and unattractive face deviants, (2) unattractive face standards and attractive face deviants, (3) attractive voice standards and unattractive voice deviants, and (4) unattractive voice standards and attractive voice deviants. Each of these four conditions contained 600 trials in total, including 450 standards (75%), 90 deviants (15%), and 60 targets (10%). The 600 trials of each condition were separated into three blocks of 200 trials each, yielding a total of 12 blocks for the whole experiment.

All trials within each of the four conditions were pseudorandomized and there were at least three standards in between two deviants. Targets (faces with red noses or voices with white noise) were always derived from standards and randomly mixed into all trials to ensure that participants attended to the stimuli. In addition, the 12 blocks were arranged in four sequences to counterbalance experimental conditions and order of presentation. Each participant completed one of the four block sequences, and each condition began with 10 practice trials. The entire duration of the experiment was about 2.5 h, including EEG preparation, practice, and experiment proper; participants rested for 1–3 min after each block.

### 2.4. EEG Recording and Pre-Processing

The EEG was recorded with a 64-channel ANT Neuro (EEGO Inc., Oberderdingen, Germany) system, with AgCl electrodes placed according to the modified 10–20 system, with CPz as on-line reference at a sampling rate of 500 Hz. Electrode impedance was kept below 5 kΩ. During recordings, a 100 Hz low-pass filter was applied. Offline, EEG signals were re-referenced to averaged mastoids and submitted to a 0.01–30 Hz band-pass filter. Ocular artifact correction was performed through independent component analysis in EEGLAB [44]. Ocular components were identified based on their topographical distribution, time course, and power spectrum. On average, 2.3 components (*SD* = 0.8) were removed per participant. Visual inspection confirmed successful removal of ocular artifacts while preserving neural signal integrity. Continuous data was segmented into stimulus-locked −200 to 800 ms epochs. Signals exceeding ±80 μV in any given epoch were automatically excluded. Other artifacts were removed according to visual inspection. All trials that immediately followed a deviant stimulus were discarded from analysis.

### 2.5. Statistical Analyses

We analyzed the vMMN for the face conditions, the aMMN for the voice conditions, and the P3 for all conditions. The vMMN was maximal over the parieto-occipital scalp in the 200–300 ms range and was measured at left and right parieto-occipital regions of interest (ROIs; electrodes P5, P7, PO5, and PO7 and P6, P8, PO6, and PO8). The aMMN was maximal over the frontal-central scalp in the 200–300 ms range and measured at left and right fronto-central ROIs (F1, F3, C1, and C3 and F2, F4, C2, and C4). The P3 was maximal over the parietal scalp in two consecutive 150 ms time windows (350 to 650 ms) and measured at left and right parietal ROIs (CP1, CP3, P1, and P3 and CP2, CP4, P2, and P4). For purposes of analysis, signals for the electrodes of a given ROI were averaged. For each stimulus modality, three-way repeated measures ANOVAs were applied to the mean amplitudes with attractiveness (attractive, unattractive), deviance (standard, deviant), and hemisphere (left, right) as factors. For all ANOVA analyses, Greenhouse–Geisser correction was applied when the assumption of sphericity was violated. Post hoc simple effects analyses were conducted with Bonferroni correction for multiple comparisons. Only adjusted *p*-values were reported.

## 3. Results

### 3.1. Behavioral Results

Participants performed with high accuracy in detecting the target stimuli (*M* = 94%, *SD* = 2%). Mean reaction time was 526 ms (*SD* = 75 ms). There was no difference in performance across conditions (all *p* > 0.05). Specifically, there was no significant difference in accuracy between face (*M* = 95% ± 2%) and voice condition (*M* = 93% ± 2%), *t* (36) = −0.45, and *p* = 0.66. Similarly, reaction times did not differ significantly between face (*M* = 537.56 ± 65.40) and voice conditions (*M* = 542.35 ± 79.98), *t* (36) = −0.21, and *p* = 0.84.

### 3.2. ERP Results

All ERP amplitude data were examined for normality using Shapiro–Wilk tests. The results indicated that all variables were normally distributed (all *p* > 0.05), satisfying the assumptions for parametric ANOVA tests.

#### 3.2.1. MMN Effect for Face and Voice Conditions

**Figure 2:**
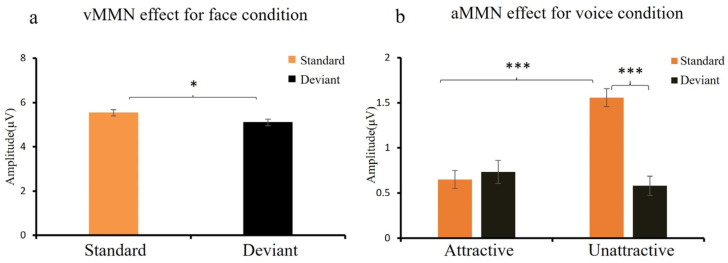
Mean amplitudes during the MMN intervals (200–300 ms). (**a**) Face condition: vMMN = visual mismatch negativity. (**b**) Voice condition: aMMN = auditory mismatch negativity. Note: * *p* < 0.05, *** *p* < 0.001.

**Figure 3:**
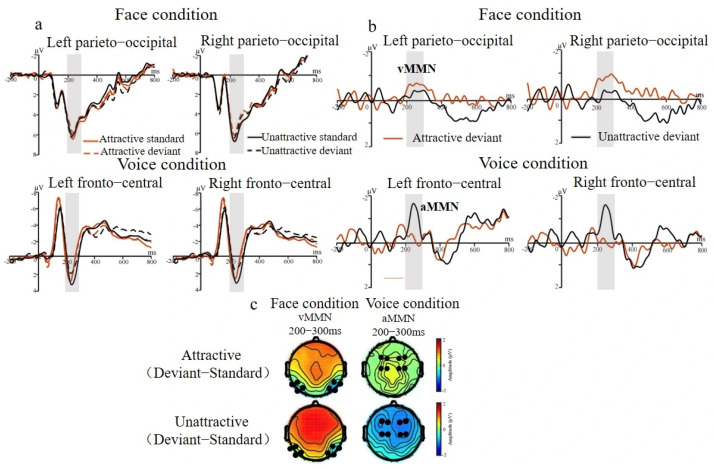
ERP results for mismatch negativities (MMN), marked by shadings. (**a**) ERPs elicited by standards and deviants in the parieto-occipital region (P5, P6, P7, P8, PO5, PO6, PO7, and PO8) in the face condition and in the fronto-central region (F1, F2, F3, F4, C1, C2, C3, and C4) in the voice condition. (**b**) Difference waveforms (MMN) obtained by subtracting standards from deviants in each condition. (**c**) Topographies of ERP differences (MMN) for attractive and unattractive oddball effects in each condition.

Figure 2 and Figure 3 illustrate the MMN effect elicited by attractive and unattractive stimuli for the face and voice conditions. In the face condition, ANOVA on mean amplitudes (200–300 ms) showed that deviants elicited larger parieto-occipital negativities than standards, *F* (1, 18) = 8.25, *p* < 0.01, *η*^2^ = 0.31, and 1 − *β* = 0.81. In addition, the interaction between deviance and hemisphere was significant, *F* (1, 18) = 10.17, *p* < 0.01, *η*^2^ = 0.36, and 1 − *β* = 0.88. Simple effect analysis indicated that deviants elicited larger negativities than standards in the right hemisphere, *F* (1, 18) = 10.48, *p* < 0.01, *η*^2^ = 0.37, and 1 − *β* = 0.90, but in the left hemisphere, this effect was marginal, *p* = 0.09 (see Figure 2a).

In the voice condition, ANOVA showed a main effect of attractiveness, *F* (1, 18) = 4.05, *p* < 0.05, *η*^2^ = 0.21, and 1 − *β* = 0.58; attractive voices elicited larger frontal-central negativities than unattractive voices. In addition, the interaction between deviance and attractiveness was significant, *F* (1, 18) = 10.04, *p* < 0.01, *η*^2^ = 0.36, and 1 − *β* = 0.88. Simple effect analysis revealed that unattractive deviants elicited larger negativities than standards, *F* (1, 18) = 15.00, *p* < 0.001, *η*^2^ = 0.46, and 1 − *β* = 0.97, but not attractive deviants, *p* = 0.81 (see Figure 2b). This interaction also revealed more negative amplitudes for attractive than unattractive standards, *F* (1, 18) = 25.53, *p* < 0.001, *η*^2^ = 0.59, and 1 − *β* = 0.99.

#### 3.2.2. P3 Effect for Face and Voice Conditions

**Figure 4:**
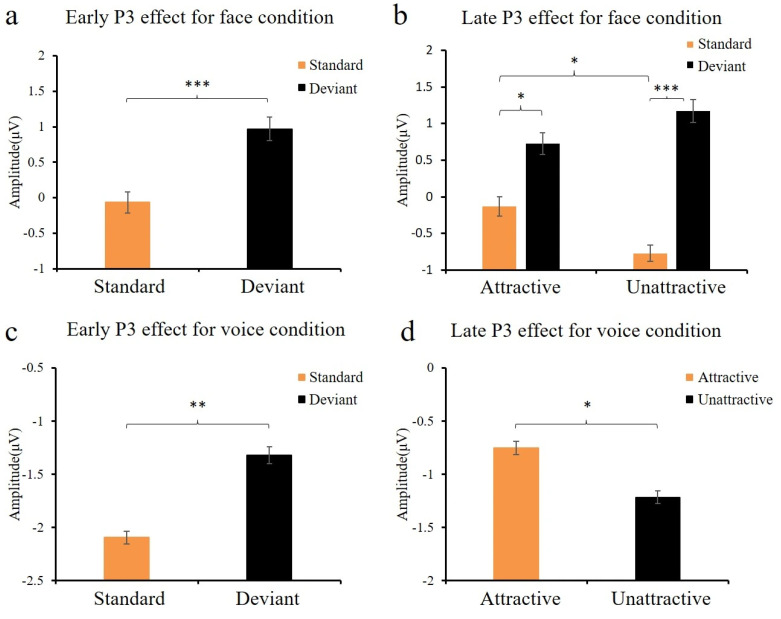
Mean amplitudes during the P3 intervals. (**a**,**b**): Face conditions, early (350–500 ms) and late (500–650 ms) segments, respectively. (**c**,**d**): Voice conditions, early (350–500 ms) and late (500–650 ms) segments, respectively. Note: * *p* < 0.05, ** *p* < 0.01, and *** *p* < 0.001.

**Figure 5:**
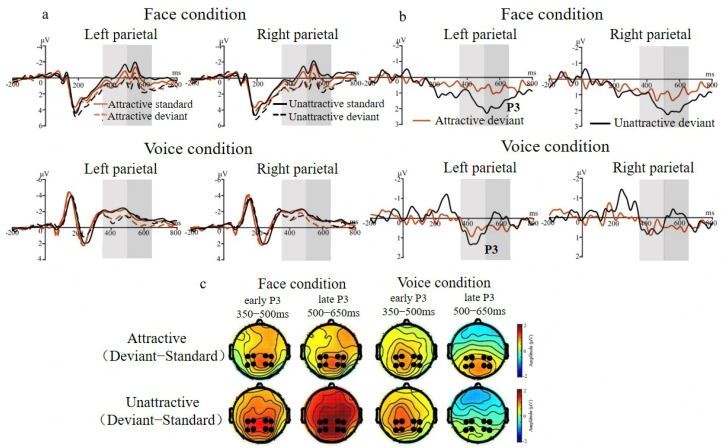
ERP results for two P3 intervals (marked by shadows). (**a**) ERPs elicited by standards and deviants over the parietal region (CP1, CP2, CP3, CP4, P1, P2, P3, and P4) in both face and voice conditions where the P3 was maximal. (**b**) Difference waveforms (P3) obtained by subtracting standards from deviants in each condition. (**c**) Topographies of ERP differences (P3) for attractive and unattractive oddball effects in each condition.

Figure 4 and Figure 5 show the ERP waveforms for the P3 ROIs elicited by attractive and unattractive stimuli for the face and voice conditions. In both conditions, the P3 was analyzed in two consecutive 150 ms time windows (350 to 500 and 500 to 650 ms). In the face condition, deviants elicited larger amplitudes than standards in both the early P3 segment (350–500 ms), *F* (1, 18) = 30.12, *p* < 0.001, *η*^2^ = 0.63, and 1 − *β* = 0.99, and the late P3 segment (500–650 ms), *F* (1, 18) = 26.22, *p* < 0.001, *η*^2^ = 0.59, and 1 − *β* = 0.99. In addition, for the late P3 segment, the interaction between deviance and attractiveness was a trend, *F* (1, 18) = 4.05, *p* = 0.05, *η*^2^ = 0.18, and 1 − *β* = 0.51. A simple effect analysis showed both more positive amplitudes for attractive deviants relative to unattractive standards, *F* (1, 18) = 4.61, *p* < 0.05, *η*^2^ = 0.21, and 1 − *β* = 0.58, as well as more positive amplitudes for unattractive deviants relative to attractive standards, *F* (1, 18) = 24.84, *p* < 0.001, *η*^2^ = 0.59, and 1 − *β* = 0.99 (see Figure 4b). This interaction also revealed larger late positivities to attractive standards relative to unattractive standards, *F* (1, 18) = 6.82, *p* < 0.05, *η*^2^ = 0.28, and 1 − *β* = 0.73, but no such difference for the deviants, *p* = 0.30.

In the voice condition, statistical analysis of the early P3 segment (350–500 ms) revealed larger positivities to deviants than to standards, *F* (1, 18) = 9.38, *p* < 0.01, *η*^2^ = 0.34, and 1 − *β* = 0.86. In addition, the interaction between deviance and hemisphere was significant, *F* (1, 18) = 4.65, *p* < 0.05, *η*^2^ = 0.21, and 1 − *β* = 0.58. Simple effect analysis indicated that deviants elicited larger early positivities than standards in both the left hemisphere, *F* (1, 18) = 12.65, *p* < 0.01, *η*^2^ = 0.41, and 1 − *β* = 0.93, and the right hemisphere, *F* (1, 18) = 6.07, *p* < 0.05, *η*^2^ = 0.25, and 1 − *β* = 0.68. For the late P3 segment (500–650 ms), the main effect of attractiveness was significant, *F* (1, 18) = 5.47, *p* < 0.05, *η*^2^ = 0.24, and 1 − *β* = 0.65, with attractive voices eliciting larger P3 amplitudes than unattractive voices (see Figure 4d).

## 4. Discussion

The present study investigated the automatic detection of facial and vocal attractiveness by assessing passive change detection for faces and voices in ‘deviant-standard-reverse’ oddball paradigms. We present ERP evidence that attractiveness information in general, but especially unattractiveness, is detected automatically. We also demonstrate that the automatic processing of facial and vocal attractiveness shows both similarities and discrepancies. Specifically, compared to high-attractive information, low-attractive deviance information induced a larger MMN effect for voices but a larger P3 effect for faces.

### 4.1. The Change Detection of Facial Attractiveness

The MMN is thought to reflect the automatic detection of deviance between sensory input and predictive memory representations generated by repeated (standard) stimuli [45]. In the present study, both attractive and unattractive faces elicited ERP components distributed over parieto-occipital regions, consistent with classic vMMN findings [30]. This observation indicates that sequentially presented attractiveness information (standard stimuli) is stored in a memory representation that facilitates predictions about what will happen next [46]. When the visual input (deviant stimuli) does not match the top-down prediction, a prediction error to the unexpected change in attractiveness reflected in the vMMN component was elicited. Since both attractive and unattractive faces elicited vMMN effects, the present experiment indicates that participants are highly sensitive to facial attractiveness changes in both directions.

Although attractive and unattractive deviant faces enhanced the P3 component, the effect was larger for unattractive deviants. The observation of bias towards unattractiveness seems consistent with the large body of evidence about a negativity bias in many domains such as emotional experience, impression formation, and intimacy [3]. Unattractive faces, which are considered to signal bad health status and/or low immunocompetence, may cause avoidance motivation. In contrast, attractive, presumably healthier faces may induce approach motivation [47]. From an evolutionary perspective, when it comes to mate choice, the key to maximizing the odds for survival and reproduction may not simply be to associate with fit individuals but also to avoid unhealthy individuals [48]. In the present study, when participants were dealing with changes in attractiveness, their motives to avoid unattractive faces may have taken precedence over their motives to approach attractive faces. According to Elliot [49], avoidant motives can focus attention on negative rather than positive situations and options and may take precedence over other goals and considerations. Meanwhile, the P3 component is concomitant with a change or updating of the stimulus representations governed by attentional processes [31]. Thus, the motives to avoid unattractive faces may have directed attentional processes more powerfully and led to stronger P3 effects in the present study than the motives to approach attractive faces.

### 4.2. The Change Detection of Vocal Attractiveness

In contrast to the symmetric pattern of vMMN elicited by deviant faces, a bias for unattractive deviants was observed in the aMMN for voices. This observation may relate to differences between perceptual representations in the auditory and visual systems. Humans characterize concrete objects (e.g., location and shape) by relying on visual perception, but “abstract objects” (e.g., melodies and sound patterns) may be characterized better in the auditory domain [50,51]. In addition, according to the model of Auditory Scene Analysis (ASA) [52], aMMN elicitation can correspond to individuals’ active exploration of alternative interpretations of the input (conveyed by top-down biasing) [50]. Consequently, in the present study, the superiority of processing ‘abstract patterns (in a ‘standard-deviant’ way) of attractiveness’ in the auditory system may cause an early-stage negativity bias, indexed by the aMMN. Alternatively, unattractive voices (e.g., a rough timbre), which are characterized by relatively simple acoustic properties as compared to attractive voices [53], may be available rather early during auditory processing and elicit an aMMN effect. In contrast, attractive voices (e.g., a smooth voice) may not stand out as saliently from an unattractive background. In contrast, when it comes to faces, attractive and unattractive facial properties may benefit from their relatively affluent information [54] and may be similarly distinguishable on a standard background of opposite attractiveness.

Interestingly, unlike the bias for unattractive deviant faces, the bias for unattractive deviant voices was not reflected in the P3 component. This may reflect that faces, compared to voices, provide richer information and require relatively more time to integrate and evaluate. These findings align with theoretical accounts of negativity bias operating across different processing stages [3]. According to the two-process model, stimulus processing occurs along a continuum from automatic to controlled [55]. The MMN component reflects automatic, pre-attentive deviance detection [29], representing the mismatch between sensory input and predictive memory representations formed by standard stimuli. In contrast, the P3 component indexes attentional resource allocation and working memory updating [31], representing more controlled processing stages. The modality-specific effects observed in the current study—whereby unattractive voices elicited stronger early automatic responses (aMMN) while unattractive faces elicited stronger late controlled responses (P3)—suggest that negativity bias may manifest at different temporal stages depending on stimulus modality. Unattractive voices eliciting MMN effects may indicate that they are automatically tagged as potential threats or unpleasant signals at the early perceptual stage. In contrast, unattractive faces triggering P3 effects may reflect that individuals invest more cognitive resources during the stages of attention and evaluation. Consequently, the current findings support the universality of the negativity bias effect and suggest that attractiveness information from different modalities exhibits processing differences in temporal dynamics.

### 4.3. Limitations and Future Directions

As a result of our investigations, suggestions may be made for future research. Firstly, researchers could utilize other approaches (i.e., the equiprobable paradigm) to control refractory effects and generalize the present findings. Secondly, distinctiveness may modulate the negativity bias observed in the vocal task. ‘Voice ratings based on vowels exhibited a moderate but significant negative correlation between attractiveness and distinctiveness’; the bias to unattractive voices may not only be induced by its lower attractiveness but also by its higher distinctiveness in the present study. Although we successfully manipulated different levels of vocal attractiveness (evidence: the main effect of attractiveness showing that attractive voices induced larger negativities than unattractive ones was observed), the potential role of distinctiveness is worthwhile for further exploration. Thirdly, the current study used a relatively small sample of 19 female participants, which limits the statistical power and generalizability of the findings. Moreover, testing only female participants viewing male stimuli restricts the generalizability to other gender combinations and populations. Future research should replicate these findings with larger, more diverse samples including male participants and explore potential gender differences in attractiveness change detection [56]. Finally, the current study’s use of static facial images may not fully capture the dynamic nature of real-world social interactions. Dynamic stimuli, such as videos depicting facial expressions or subtle movements, could offer more ecologically valid measures of attractiveness processing [57,58]. Future research should employ dynamic facial stimuli to investigate whether motion and temporal cues modulate the negativity bias and automatic change detection mechanisms observed in the current study.

## 5. Conclusions

Voices or faces deviating in attractiveness were robustly detected automatically but differed in time course, depending on valence and modality. Specifically, detecting attractive items was weaker than detecting unattractive ones, demonstrating a negativity bias; this bias was present in the early stages of processing voices and in the late stages of processing faces. We speculate that—at least in females, as tested here—this negativity bias in (un)attractiveness detection may serve greater importance to avoid social partners with unfavorable health or immune status than to approach those with apparently good status.

## Data Availability

The data presented in this study are available on request from the corresponding author due to privacy.
